# A Care Delivery Model of Temporary Transfer of Medical Workers and Equipment to Confine a Pandemic

**DOI:** 10.3389/fmed.2020.561864

**Published:** 2021-02-03

**Authors:** Han Zhang, Danhui Yang, Min Yang, Liucun Li, Hong Luo, Ata Murat Kaynar

**Affiliations:** ^1^Department of Respiratory and Critical Care, The Second Xiangya Hospital, Central South University, Hunan, China; ^2^Research Unit of Respiratory Disease, Central South University, Hunan, China; ^3^Hunan Diagnosis and Treatment Center of Respiratory Disease, Hunan, China; ^4^Clinical Research, Investigation, and Systems Modeling of Acute Illness (CRISMA) Laboratory, Department of Critical Care Medicine, University of Pittsburgh School of Medicine, Pittsburgh, PA, United States; ^5^Department of Anesthesiology and Perioperative Medicine, UPMC Presbyterian, Pittsburgh, PA, United States

**Keywords:** COVID-19, SARS-CoV-2, medical team delivery, respiratory care, pandemic

## 1. Introduction

Since December 2019, the coronavirus disease 2019 (COVID-19) caused by severe acute respiratory syndrome-coronavirus-2 (SARS-COV-2) virus reached pandemic levels (1). As of March 1, 2020, there are 79,968 confirmed cases in China, and among these confirmed cases, 2,873 deaths occurred (2). Once the COVID-19 develops to critical illness, the mortality rate will rise drastically (3, 4). Among critically ill COVID-19 patients, 63.5% were treated with high-flow nasal cannula though controversial, 71% with mechanical ventilation, 11.5% with prone position ventilation, 11.5% with extracorporeal membrane oxygenation (ECMO), and 17% with continuous Renal Replacement Therapy (CRRT) at Wuhan Jin Yin-tan hospital (Wuhan, China) resulting in an unprecedented surge in critical care resource utilization (4). Similarly, most countries encountered this challenge since the outbreak of COVID-19 throughout the world, resulting in an urgent need of sharing the successful anti-plague strategies between different countries.

## 2. Imbalance of Medical Resource in Hunan

As one of the provinces closest to the Hubei, the epicenter of China, Hunan province has adopted bundled care delivery model strategies to prevent the spread of the COVID-19, including a model of medical team delivery to the sites with increased disease burden due to the regional difference in economic development, number of beds, physicians, nurses, respiratory therapists, and other medical personnel (5, 6). The Chinese hospital network is built on a multi-layered hub-and-spoke model (7, 8). According to the number of beds, departments, medical personnel, equipment, and expertise, hospitals in China are classified as primary, secondary, and tertiary hospitals (9). A primary hospital is a typical township hospital with <100 beds focusing on primary care. Secondary hospitals are located within medium sized cities, counties, or districts and contain between 100 and 500 beds providing comprehensive health services. Subsequently, tertiary hospitals with more than 500 beds provide specialist health services and they serve as medical hubs providing care to multiple regions.

In Hunan, the province next to the COVID-19 epicenter Wuhan, most tertiary hospitals are located in the capital city Changsha. In Changsha, there are nine tertiary hospitals and except Hengyang city, all other cities have one or no tertiary hospitals. The data are showing distribution inequality, especially the access to tertiary care hospitals within the Hunan province (Table 1) (10, 11). As a result, many hospitals located in small towns or prefecture-level cities do not have the infrastructure and staffing conditions to provide high-quality treatment for COVID-19 patients. As such, facing the problem of the regional imbalances of medical resources, most severe patients in primary and secondary hospitals could be transferred to higher-level tertiary hospitals. Along the lines of hub-and-spoke system of any healthcare enterprise, centralized care leads to more efficient utilization of scarce resources such as intensive care and improved clinical outcomes (12). However, the benefits provided by a centralized care model needs to be balanced against the risk of infection transmission due to inter-hospital transfer, delay in access to intensive care, and loss of expertise in peripheral hospitals (12). Especially due to its high transmissibility, inter-hospital transfer strategies are challenging during the COVID-19 pandemic (13, 14). Thus, it would be reasonable to reduce the number of transfer processes once a COVID-19 is identified.

**Table 1:** The number of tertiary hospital, beds, medical workers, physicians, and nurses in each city of Hunan province.

**City**	**Population (10 K)**	**Confirmed cases**	**Tertiary hospital**	**Beds**	**Medical workers**	**Physician**	**nurse**	**Tertiary hospital/100 k**	**Bed/100 k**
Changsha	815.47	242	9	77,253	81,548	30,793	38,727	0.110	947.3
Xiangtan	286.48	36	1	19,336	19,508	7,530	8,765	0.035	674.9
Zhuzhou	402.08	80	1	26,811	26,616	10,534	12,003	0.025	666.8
Yueyang	579.71	156	1	35,172	31,595	15,187	11,324	0.017	606.7
Changde	582.72	82	1	39,679	35,750	17,387	13,198	0.017	680.9
Chenzhou	474.45	39	1	33,485	29,669	11,693	13,051	0.021	705.7
Hengyang	724.34	48	4	46,537	42,260	17,936	17,352	0.055	642.4
Yiyang	441.38	60	1	28,141	24,964	11,167	9,876	0.023	637.5
Loudi	393.18	76	1	20,138	21,127	9,484	8,133	0.025	512.1
Zhangjiajie	153.79	5	0	10,838	9,140	3,303	3,949	0	704.7
Yongzhou	545.21	44	1	40,794	32,165	13,656	12,908	0.018	748.2
Huaihua	497.96	40	1	35,033	31,258	12,005	13,355	0.020	703.5
Shaoyang	737.05	102	1	43,760	35,224	14,478	14,536	0.014	593.7
Xiangxizhou	264.75	8	0	14,457	16,741	5,817	7,207	0	546.1

## 3. Anti-COVID Strategy Adopted in Hunan

Aligning with the strategy of reducing the transfer of COVID-19 patients, Hunan Health Commission formulated a strategy to facilitate the treatment of COVID-19 patients at the prefecture-level cities instead of transfers. This is learned from the valuable experience of the fight against SARS virus in 2003. At that time, China also set up dedicated doctors and nurses led by experienced respiratory and infectious disease experts in various provinces to provide special care for all patients admitted to designated wards (15). Hunan Province has carried out very strict quarantine inspection (16) and isolation measures. Through timely identification, isolation, and active and effective treatment, the epidemic situation can be controlled. Until the end of the SARS epidemic, there were only seven SARS patients in Hunan Province, and all of them were cured. During the current pandemic, improvements were made on the basis of the previous experiences. The core idea of this strategy was to assign a medical “anti-COVID team” to each city of Hunan, resulting in a total of 14 teams being deployed. Each “anti-COVID team” consisted of experts from the critical care medicine, pulmonary and critical care medicine, infectious diseases, and emergency medicine departments from the tertiary hospitals of Changsha, the capital city of Hunan. Besides the “anti-COVID team” for each city, there are two additional teams “airway management team” and “ECMO team” shared by all cities in Hunan. The “ECMO team” experts are in charge of ECMO management. The “airway management team” was established with four respiratory therapists (RT) experienced in the management of emergency airways. Once there is an urgent need for RT or ECMO operations in a city, members of these two teams will be mobilized and transferred to the city to aid the treatment of COVID-19 patients. The urgent need is notified by the “anti-COVID” team dispatched to the designated hospital and then transferred to Hunan Health Commission that is responsible for deploying the members of “airway management team” and “ECMO team.” For example, Loudi is a prefecture-level city of Hunan and has a population of 3.87 million. As of March 11, 2020, the Center Hospital of Loudi, the only designated hospital to treat COVID-19 patients in Loudi, have received 76 confirmed cases, including five severe illness and two critically ill patients (17). Those two critically ill patients needed invasive mechanical ventilation, prone position and ECMO. However, in Hunan province, only four tertiary hospitals, namely the Xiangya hospital of Central South University, the second Xiangya hospital of Central South University, the third Xiangya hospital of Central South University, and Hunan Provincial People's Hospital, have professional RTs. As a result, physicians or nurses mainly perform mechanical ventilation in the Center Hospital of Loudi without training in respiratory care or mechanical ventilation, which brings many challenges to the treatment of the patients. Even worse, the Center Hospital of Loudi does not have completed isolated rooms. Patients of Covid-19 are separately isolated in temporary rooms of 10 m^2^. This hospital is also confronted with problems of lacking medical equipment (e.g., high-flow nasal oxygen and ECMO). Thus, in order to provide high-quality treatment for two critically ill patients in Loudi, besides the “anti-COVID team,” the Hunan Health Commission further mobilized an RT from the “airway management team” and two ECMO experts from the “ECMO team” to Loudi. In addition, the transferred medical personnel are also responsible for training the physicians and nurses, who work in the designated hospital with diagnosis, treatment, respiratory care, and ECMO. With the efforts of transferred and local medical personnel, there was no death case in Loudi city caused by COVID-19 disease as of December 18, 2020. The mortality rate of COVID-19 cases in Hunan Province was <0.4%, while the overall mortality rate of COVID-19 patients in all provinces except Hubei was about 0.6%. In Hubei Province, as the first place of the epidemic in China, the total mortality rate of COVID-19 confirmed patients in Hubei Province reached 6.6% due to the darkest time in the early days of the epidemic.

The case of assigning Loudi with “anti-COVID team” from provincial capital was not specific to Hunan, but part of China's anti-epidemic strategy. This was a different approach compared to other countries with an emphasis on models, where patients were transferred to expert centers (18). The “peripheralized care deliver model” can help underdeveloped regions to access the rich medical resources of developed regions within a country.

## 4. Discussion

While the care delivery model mentioned above is exciting and now tested during a pandemic, there were also many problems. First of all, this model would not have worked if the tertiary hospitals were overwhelmed with a surge of patients preventing sharing the experts to peripheral hospitals. This model also unmasked the scarcity of limited qualified personnel. Interestingly, there are approximately 140 RTs participating in the “anti-COVID teams” across the China, which highlights the need to train personnel, who can manage ventilators. Respiratory therapy has become a formal career in China only in 2019 and there are no official certification or license for RTs at this moment (19). In addition, only one university has provided degree in respiratory therapy. There are a total of 259 students in respiratory therapy, who graduated from the university since 2004. Unexpectedly, 31% of these graduates engaged in other jobs rather than respiratory therapy (20, 21). Besides this university, there are only two other colleges providing short-term training program for physicians or nurses. As a result, almost all primary and secondary hospitals and even some tertiary hospitals do not have professional RT. The tasks of RTs in these hospitals are mainly conducted by physicians and nurses highlighting the need for increased efforts devoted to the education of respiratory therapy in China. In addition, the success of the care delivery model presented in this paper benefits a lot from the medical equipment transferring between tertiary hospitals and peripheral hospitals, which could lead to a threat of running out of medical equipment when there are many critical cases. Moreover, the transferring of medical equipment is also harmful to the efficiency of the care delivery model due to the time costs on the disassembling, transporting, installation, and testing. Thus, it is better to add equipment infrastructure in the various peripheral locations, allowing the deployed teams to be immediately operational when another pandemic happened in future.

In summary, the care delivery model in China during the COVID-19 pandemic was creative and successful within that country and its regulations. The transfer of healthcare personnel between cities and even hospital systems would require regulatory adjustments for such a system to be adapted in other countries.

## Author Contributions

HZ and DY prepared the draft of the commentary under supervision of HL and AK. HL and AK revised the manuscript. MY and LL collected the data used in the paper. All authors contributed to the article and approved the submitted version.

## Conflict of Interest

The authors declare that the research was conducted in the absence of any commercial or financial relationships that could be construed as a potential conflict of interest.
